# Delusional Parasitosis in Comorbidity With Shared Paranoid Disorder in a Marriage

**DOI:** 10.1155/2024/6728600

**Published:** 2024-09-26

**Authors:** Jonatan Escobar-Herrera, Gibran Raymundo León-Gallegos, Omar E. Valencia-Ledezma, Rafael García-Rascon, Nicolás Santiago-González

**Affiliations:** ^1^ Unidad de Psiquiatría del Hospital Regional de Alta Especialidad de Ixtapaluca Instituto Mexicano de Seguro Social Para el Bienestar (IMSS-BIENESTAR), Ixtapaluca, Mexico; ^2^ Unidad de Investigación del Hospital Regional de Alta Especialidad de Ixtapaluca Instituto Mexicano de Seguro Social Para el Bienestar (IMSS-BIENESTAR), Ixtapaluca, Mexico

**Keywords:** case report, delusional parasitosis, ekbom syndrome, folie à deux, shared paranoid disorder

## Abstract

Delusional parasitosis is a psychotic disorder where the patient has the delusion of being infested with some insect or parasite. In contrast, shared paranoid disorder or folie à deux is described when the same delusions affect two or more closely related people. It is common for these two situations to cause comorbidity in the family unit. This case report concerns a couple married for 37 years. The husband described that 2 years ago, he began with a tingling sensation throughout his body, related to the presence of parasites coming out from all his body orifices, with no evidence of self-harm. Likewise, the wife reported symptoms of formication and the feeling that there were invisible animals, as mentioned by her husband, and that she felt the parasites running throughout her body. The husband was diagnosed with endoparasitic delusional parasitosis, which caused folie à deux in his wife due to ectoparasitic parasitosis. The patient's treatment included sertraline and risperidone in oral dosage lasting 3 months reducing delirium, later biperidene was prescribed due to main treatment's side effects such as akathisia and sialorrhea, however the patient could not take the medication due to economic reasons. The wife was asked to sleep in a separate room, and she reported that the sensory hallucinations disappeared as soon as she slept in a different room. We conclude that the pharmacological approach, the intervention in the family life, and the gradual reintegration of marital habits once the patient improves are crucial in the therapy of delusional disorder.

## 1. Introduction

Delusional parasitosis or Ekbom syndrome (ES) is a disorder where the patient believes to be infested by insects or some parasite [1] and closely related people are at risk of developing a shared paranoid disorder of delusional infestation known as folie à deux [2].

Parasitic delirium also known as ES is relatively more common in white population, around 20 cases in one million per year and proportionally 80 cases per million in general population [3], in the other hand there is a lack of precise information about the exact prevalence of this shared-psychiatric disorder. While it is equally presented in both, women and men, there is a tendency in women to experiment more shared-psychotic symptoms in comparison to men.

It is key to differentiate ES from insects or parasite's phobias, not involving fear to this agents in ES. Also not confusing it with Wittmack–ES or restless legs syndrome. ES affects all-aged patients with uniform distribution between genders before reaching 50 years old, then women presents higher prevalence, 2 : 1 ratio. Incidence is bimodal reaching two peaks between 20–30 years old and after 50 years old [4, 5].

Symptoms may last up 13 years being itching or pruritus is the most common symptom. As the mental disturbance progresses, visual hallucinations may arise as well as scratching and elimination behaviors become increasingly unusual and ritualistic over time. Gradually these symptoms dominate the patient's life style and thinking, significantly affecting the diary behavior and well-being [5].

In relation to shared psychotic disorder based on the Diagnostic and Statistical Manual of Mental Disorders (DSM−5), induced psychosis is a complete separated entity included in schizophrenia spectrum and other psychotic disorders [6]. The International Diseases Classification (CIE−10) categorizes this disease as a delusional disorder instead of a separated entity [7]. Our case report presents the comorbidity between delusional parasitosis and shared paranoid disorder in a marriage.

## 2. Case Presentation

The patient's information includes two individuals married for 37 years living in a rural area with dogs and cats (not vaccinated or dewormed) who reported the presence of mice in the house. The primary patient was the husband, considered the “inducer.“ He was a 60-year-old male dedicated to business, with a history of alcoholism suspended 13 years ago. The secondary patient, the “inductee,” was the wife, a stay-at-home 55-year-old female.

During the mental health assessment, the patient (husband) was cooperative, attentive, and slightly nervous answering the questions asked by the psychiatrist. He showed signs of anxiety when mentioning his symptoms although his emotional reaction and construction of ideas were adequate talking about other topics such as economic condition, work activities, and the relationship with each member of his family. No course of thought alterations were detected but a hypochondriac somatic delusional ideation, expressing a belief of external and internal infestation by “bugs”. No obsessive ideas, phobias, or passive phenomenon were found. The patient expressed great concern about his physical status and mentioned some experiences compatibles to generalized cenesthetic hallucinations, he reported having a tingling sensation all over his body for 2 years, which he associated with the presence of parasites. He said: “It comes out where the poop comes out, from my eyes, my nose, and my genitals” sic pac. Intelligence evaluated as average while judgement and reasoning involving logical conclusion making were compromised. His main objective was “get rid of bugs as soon as possible”.

Upon extensive examination of the integuments, no dermatological lesions or scrapes were found. Physical examinations and normal laboratorial test results allowed ruling out organic diseases as cause. Nevertheless, additional test could not be performed such as cranial magnetic resonance imaging or toxicological test to rule out used of pesticides, information only corroborated by clinical interview.

The diagnostic evaluation was associated with primary delusional parasitosis of endoparasitic location, and through the evolution of his disease, he began to share his symptoms with the person closest to him, in this case, his wife, who had been his life partner. Thus, it was identified as a shared paranoid disorder or Foliè a deux. Meanwhile the wife states that at the beginning she performed an exhaustive cleaning in their house, bedroom, and in all the places her husband carried out any activities such as resting. In which there was no indication of bugs or any animal that could be responsible for her husband's symptoms, leading her to the conclusion of what her husband claimed was not real and probably had a hidden intention. As her husband's parasitization feeling and scratching behavior worsened, she begins to present formication symptoms lasting 1 year with less intensity but similar to those of her husband, claiming that “I feel that there are invisible animals that he tells me, and I feel that they run all over my body, I do not see them, but I feel them”, sic pac. Leading to be diagnosed with primary ES with ectoparasitic presentation, in consequence, Foliè a deux.

It is important to consider that at the beginning, the patient's wife reported that she did not make sense of her husband's description of the origin and nature of the “parasites” he mentioned having and that on several occasions, she thoroughly cleaned her house in front of her husband to calm his anxiety. She also tried home remedies to relieve symptoms, she described as a sensation of tingling and itchiness but was unsuccessful. Likewise, none of their three children who live with the patient had presented any symptomatology related to the delirium of parasitosis.

The husband's therapeutic intervention began with selective serotonin reuptake inhibitors to treat the anxiety symptoms and antipsychotics at regular doses. The medication of choice was sertraline, the patient was instructed on the correct way to start the treatment, which had to be taken orally. Risperidone was administered, and following up after 15 days. The wife and family members were asked to sleep separately to improve the treatment response.

The results observed in the follow-up visit at the clinic were that after 3 months of treatment, the patient responded favorably. He mentioned that the feeling of parasitism decreased and reported that his quality of life had improved after starting the pharmacological treatment. Monthly follow-up appointments to evaluate continuously the treatment response was necessary to check up symptom evolution on antipsychotic drugs and to determine whether the patient experiences significant sustained improvement.

Later follow-up revealed mild akathisia and sialorrhea prescribing biperidene to ease the aforementioned symptoms, however due to economic reasons the patient could not take the medication, even more, by own decision the patients decreased the risperidone dosage resulting in mild parasitic ideation return.

In the wife's case, the sensory hallucinations or delusions of parasitism disappeared immediately as soon as she began sleeping in another room, without having returned even when the husband again presented these delusional ideas. The reintegration of the family nucleus and conjugal habits were gradually resumed once the patient improved.

## 3. Discussion

This is a clinical case of delusional parasitosis in comorbidity with paranoid disorder shared in marriage, a rare condition with little data on the Mexican population, which makes this case relevant.

Shared delirium occurs between two people called the inducer and the inductee, whose relationship is very intimate [8]. The inducing patient (husband) expressed delusional parasitosis, a disorder included within the spectrum of schizophrenia in the DSM-5 that applies when the central theme of the delirium involves bodily functions or sensations [9]. The delirium of infestation is not limited to parasites only but encompasses different organisms [10]. Since no other psychiatric or neurological symptoms were identified, primary delusional parasitosis was diagnosed. Also, the patient stated that the organisms were coming out from the body orifices. According to the site of location, three subtypes have been described: ectoparasites (skin), endoparasites (internal organs or orifices), and mixed [5]. In delusional infestation, the patient has a false and fixed idea of being infested by small living or inanimate pathogens, with no objective evidence [11].

The main symptom presented was tingling, with no evidence of self-harm. Characteristic symptoms include pruritus, pain, burning, and the “matchbox” sign in which the patient brings small body parts (hair, scabs, nails, skin scales, among others) in a container to the treating physician, which is considered evidence of injurious agents [12]. It should be considered that the patient may feel misunderstood or sometimes mocked, as for him or her, the existence of these parasites is real, and no argument will be valid or sufficient to convince the patient otherwise, no matter how much the inexistence of the parasites is analyzed under a logical framework and even when the clinical evidence does not justify their existence [13].

To carry out a precise approach it is necessary to consider other medical conditions that allow making a differential diagnosis that present similar symptoms such as scabies, dermatitis herpetiformis, insect prurigo, hallucinations, obsessive–compulsive disorder, and other chronic psychoses [3]. This conditions also resembles Morgellons' disease, such characterized by microscopic fibers embedded within the skin and in some cases might be part of a shared psychosis [14]. It is important to performed exhaustive analysis and test to reveal the underlying cause to the parasitic ideation.

On the other hand, the wife was diagnosed with ectoparasitic ES. The way in which the patient's wife shared this delusion is emphasized since, for this to happen, there must be both a close relationship and a submissive relationship to the delusional ideas of the person suffering from the delusion [15]. The previous highlights the importance of reviewing the family unit and restructuring the family dynamics simultaneously, which is why the directive to separate the wife from nocturnal activities such as sleeping together was included in the care plan. This measure was of great support both for the suffering of the wife and for the patient, as a source of reaffirmation of the ideas of parasitism was eliminated.

Comorbidity with shared paranoid disorder, or folie à deux, is a rare disorder characterized by the presence of psychotic symptoms, usually delusions, in two or more closely related people, typically relatives, where only one of the patients presents with an actual psychotic disorder [16]. It must be noted that for a shared psychotic disorder to be possible, there must be a hierarchy or a perception of superiority of the person who develops this disorder. The latter allowed the wife to be identified as the passive subject, who was able to resist for at least a year the pressure that her husband exerted on her to convince her that her pathology had a real origin. Eventually, her efforts were surpassed, and finally, after some resistance, she ended up intervening in the delirium, rectifying, modifying, coordinating, and making it even more tangible and credible. It has been observed that the more the start of treatment is delayed, the more delusional the patient becomes [5].

Regarding to prognosis and treatment, it is suggested to continue the treatment two up to 6 months after all symptoms disappear to ensure a complete recovery [17]. Approximately half of patients archive total symptom remission. No statistical difference was found in antipsychotic treatment with first or second-generation drugs. It is prioritize to start treatment as soon as possible since the longer the time from the onset of symptoms to treatment the worse the prognosis will be for the patient. Regarding prognosis and treatment, it is essential to mention that each person may respond differently to treatment due to physiological predisposition or comorbidities. Therefore, it is necessary to take a comprehensive approach to the condition [10]. How the benefits of treatment are approached, and the focus on the integrity of the patient improves adherence and prognosis, which undoubtedly benefits healthcare systems and patients [18]. Therefore, this case report will serve as a reference for managing this comorbidity in the Mexican population.

## 4. Conclusion

Endoparasitic delusional parasitosis of the inducing patient was treated with sertraline and risperidone. Presenting side effects of akathisia and sialorrhea, by own decision the patient decided to reduce the dose of risperidone, resulting in the return of the symptoms, although with less severity. Due to the close coexistence with his wife, she developed ectoparasitic ES in comorbidity with shared paranoid disorder. In this clinical case, the wife experienced an improvement in psychotic symptoms since she managed to become aware of her conditions (insight). It was not require to start antipsychotic medication but behavioral monitoring to maintain control of symptoms and prevent relapses that was archive by sleeping in a separate room from the husband. Therefore, we conclude that the pharmacological approach, the intervention in family life, and the gradual reintegration of marital habits once the patient improves are crucial in the treatment of delusional disorder.

## Data Availability

The case report data used to support the findings of this study are included within the article.
